# Supplementary material for: Winter temperatures limit population growth rate of a migratory songbird
Source: Nat Commun. 2017 Mar 20;8:14812. doi: 10.1038/ncomms14812 (PMC5364383; doi:10.1038/ncomms14812)
Supplement: Supplementary Information — Supplementary Figures, Supplementary Methods and Supplementary References [file ncomms14812-s1.pdf]

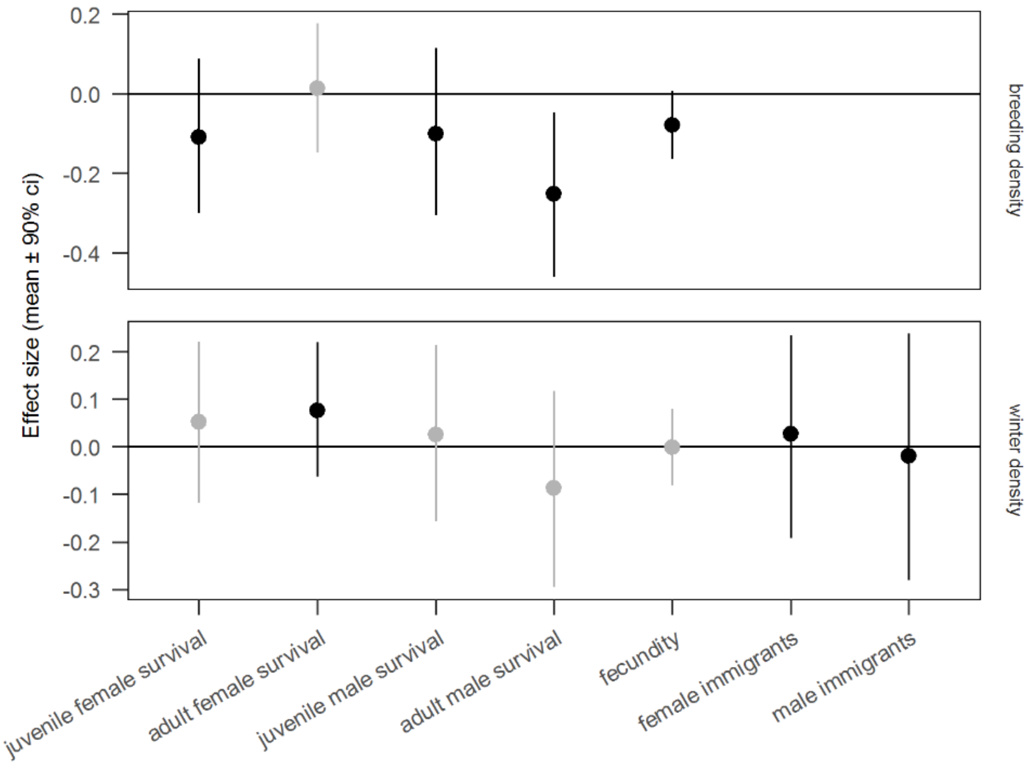

Supplementary Figure 1. Effects of density at the breeding and wintering grounds on vital rates of a breeding population of Savannah sparrows on Kent Is., NB, Canada. Vital rates were modeled against standardized densities at the breeding and wintering grounds within an integrated population model. For a given vital rate, the strongest effect is shown in black and the weaker effect is shown in grey. Survival probabilities from year *t* to *t+1* were regressed against standardized breeding population density in year *t* and fecundity in year *t+1* was regressed against breeding population density in year *t+1*. All vital rates in year *t* were regressed against population density at the wintering grounds from the previous winter (counts occurred in late Dec of year *t-1* or early Jan of year *t*). The strongest effect for each vital rate is shown in black. Density-dependence was strongest for adult male survival and fecundity. Both vital rates were negatively correlated with population density at the breeding grounds (adult male survival = -0.25 [**95% CI =** -0.47, -0.05]; fecundity = -0.08 [**95% CI =** -0.17, 0.01]. Adult female survival was the only vital rate for which the effect of winter density was greater than that of breeding density.

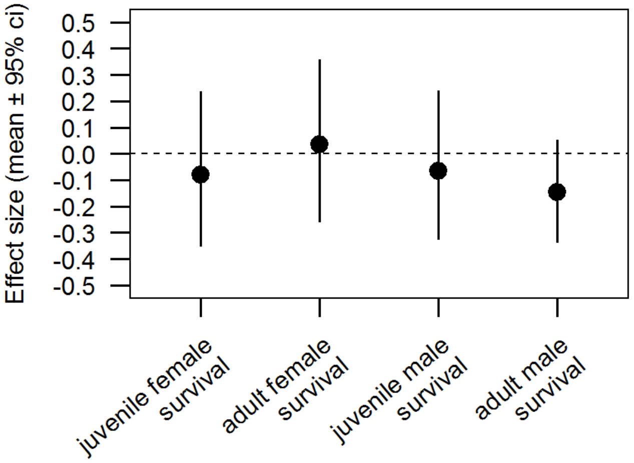

Supplementary Figure 2. Interactive effect of breeding density and subsequent winter temperature on survival of Savannah sparrows from a breeding population on Kent Is., NB, Canada. Annual apparent survival probabilities were modeled against standardized average daily mean temperature and precipitation as well as population densities at the breeding and population-specific wintering grounds within an integrated population model. Effects of the interaction between breeding density and subsequent winter temperature on survival were weak for both sexes and age groups (adult male = -0.15 [95% CI = -0.34, 0.05], juvenile male = -0.06 [95% CI = -0.33, 0.24], adult female = 0.04 [95% CI = -0.26, 0.36], juvenile female = -0.08 [95% CI = -0.35, 0.24]).

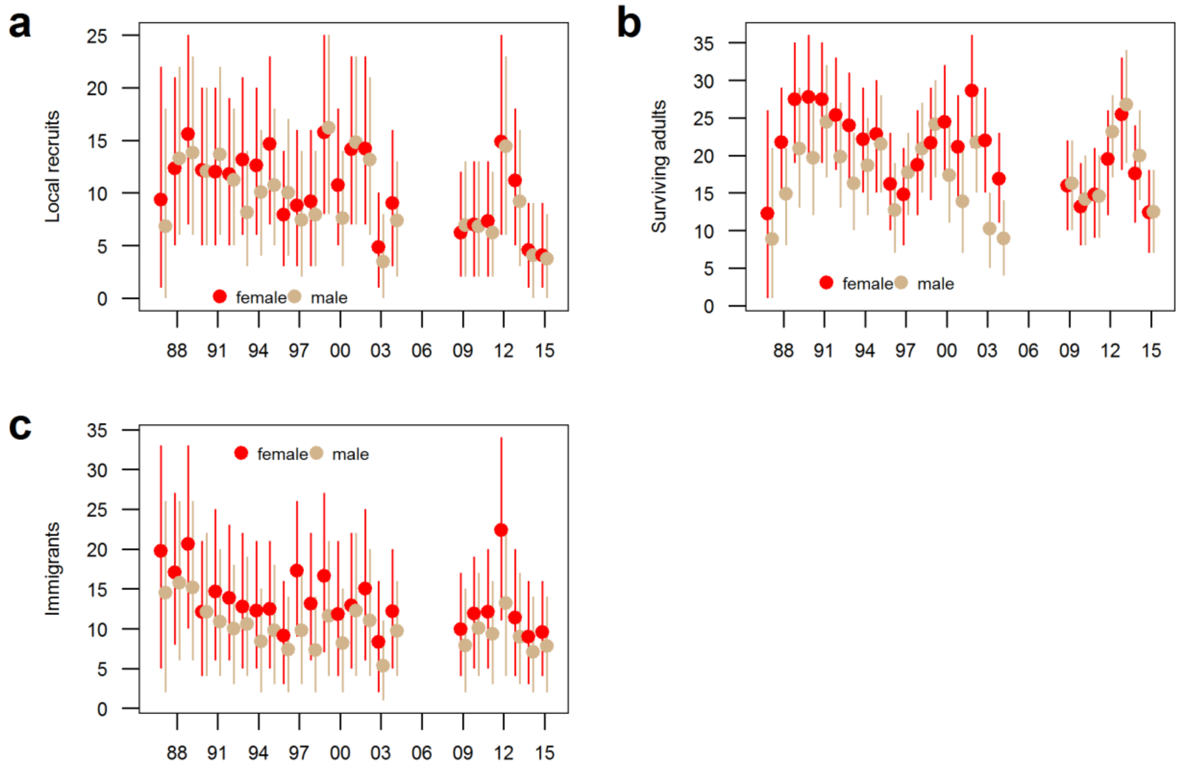

Supplementary Figure 3. Estimates of population size and stage- and sex-specific abundances for a breeding population of Savannah sparrows on Kent Island, NB, Canada. (**a-c**) Estimates (mean ± 95 credible interval) of population structure were obtained by jointly analyzing mark-recapture/resighting, reproductive success, and population count data using an integrated population model.

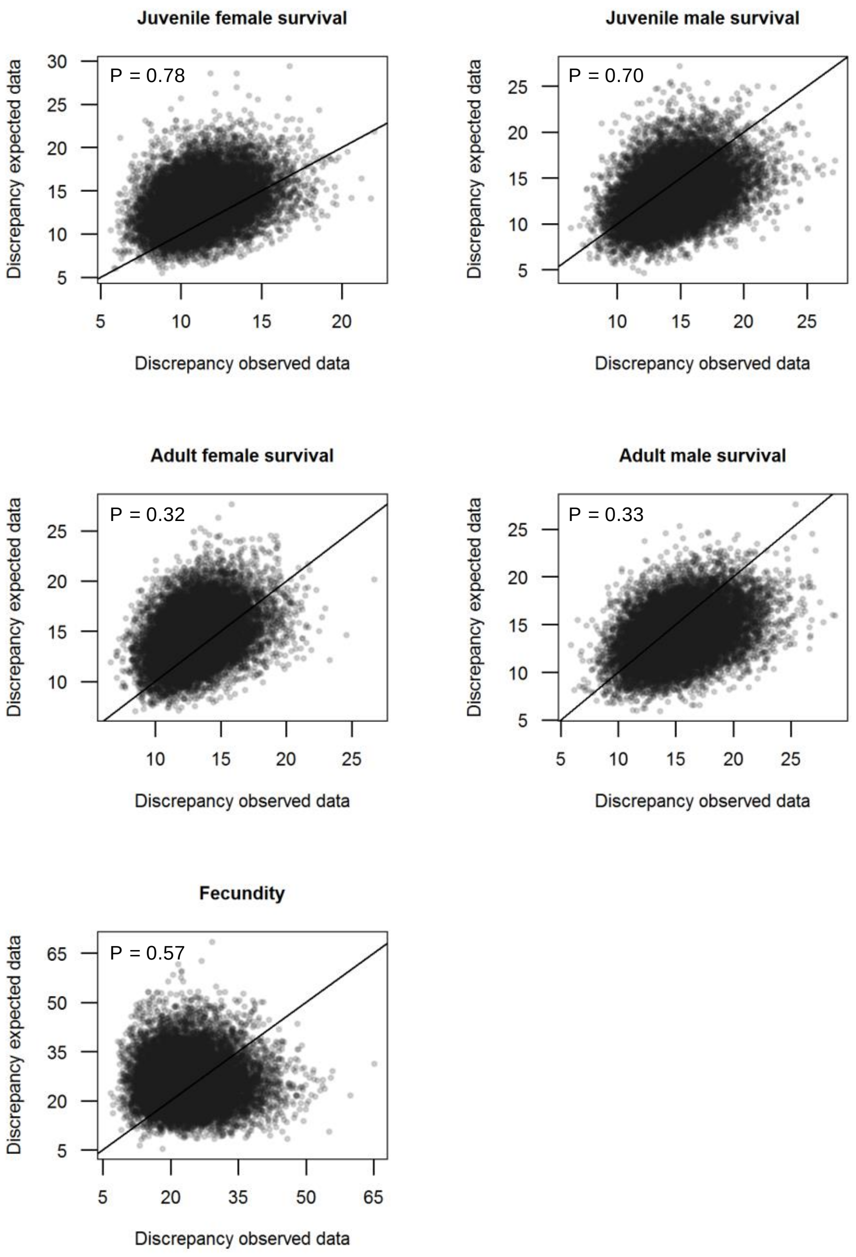

Supplementary Figure 4. Posterior predictive checks of model fit for mark-recapture/resighting and reproductive success data. Scatterplots show the discrepancy between observed and expected data simulated from Cormack-Jolly-Seber and Poisson models for the mark-recapture/resighting and reproductive success data, respectively. A model that fits the data perfectly will result in a Bayesian p-value = 0.5, corresponding to equal numbers of points above and below the 1:1 line. P-values greater than 0.5 indicate that the expected data are more variable than the observed data, whereas p-values < 0.5 indicate that expected data are less variable than the observed data.

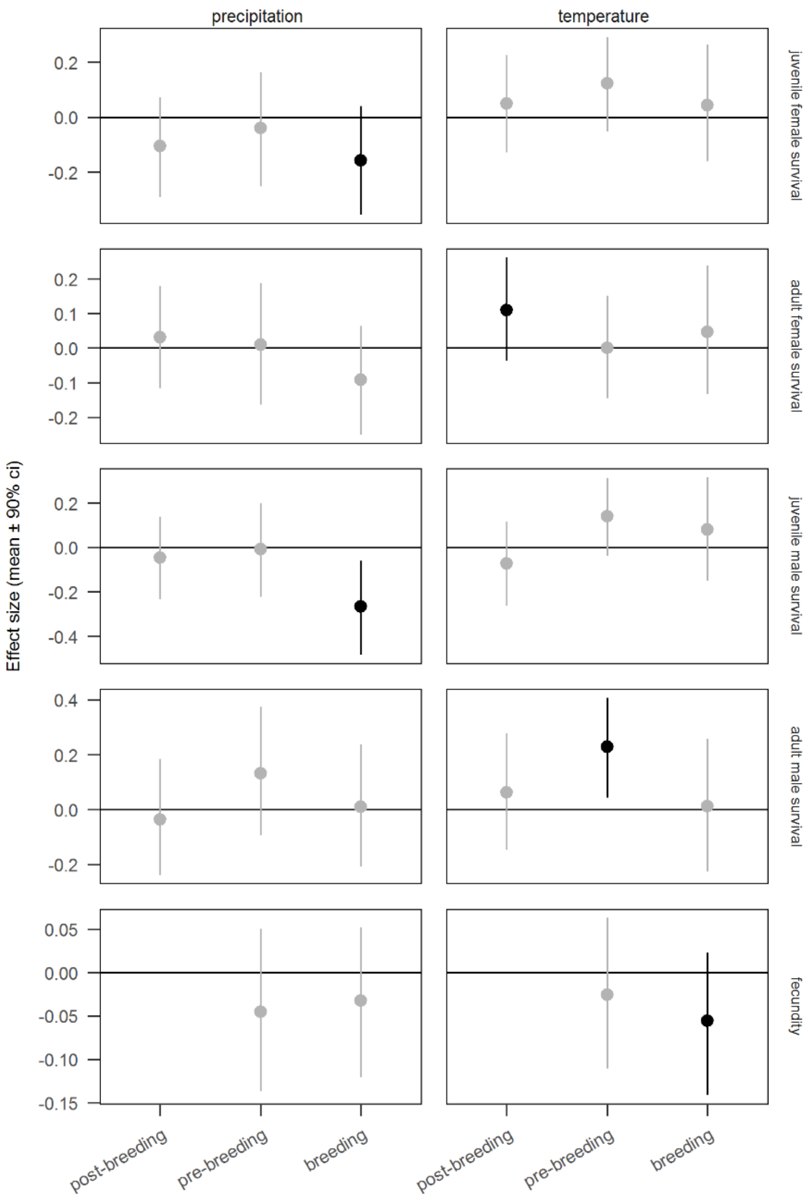
precipitation
temperature
juvenile female survival
adult female survival
juvenile male survival
adult male survival
fecundity
Effect size (mean ± 90% ci)
0.2
0.0
-0.2
0.1
-0.1
-0.4
0.4
0.05
0.00
-0.05
-0.10
-0.15
post-breeding
pre-breeding
breeding

Supplementary Figure 5. Effects of weather at the breeding grounds on vital rates of Savannah sparrows on Kent Is., NB, Canada. Vital rates were modeled against standardized average daily mean temperature and precipitation during the pre-breeding, breeding, and post-breeding periods within an integrated population model. For a given vital rate, the strongest effect is shown in black and weaker effects are shown in grey. Survival probabilities from year *t* to *t+1* and fecundity in year *t+1* were each regressed against average daily mean temperatures during the pre-breeding and breeding periods in year *t+1*. Survival probabilities from year *t* to *t+1* were also regressed against average daily mean temperature and precipitation during the post-breeding season in year *t*. Precipitation during the breeding period had the strongest effect on juvenile survival of both sexes (juvenile female = -0.16 [**95% CI =** -0.35, 0.04]; juvenile male = -0.27 [**95% CI =** -0.48, -0.06]), temperature during the post-breeding period had the strongest effect on adult female survival (0.11 [**95% CI =** -0.04, 0.26]), and pre-breeding temperature had the strongest effect on survival of adult males (0.22 [**95% CI =** 0.04, 0.41]). Juvenile survival of both sexes was negatively correlated with breeding precipitation, whereas survival probabilities of adult males and females were positively correlated with pre-breeding and post-breeding temperatures, respectively. Weather at the breeding grounds was most weakly correlated with fecundity, with temperature during the breeding period having the strongest effect (-0.05[**95% CI =** -0.14, 0.02]). We did not evaluate effects of weather during the post-breeding season on fecundity, nor did we consider effects of weather at the breeding grounds on immigration.

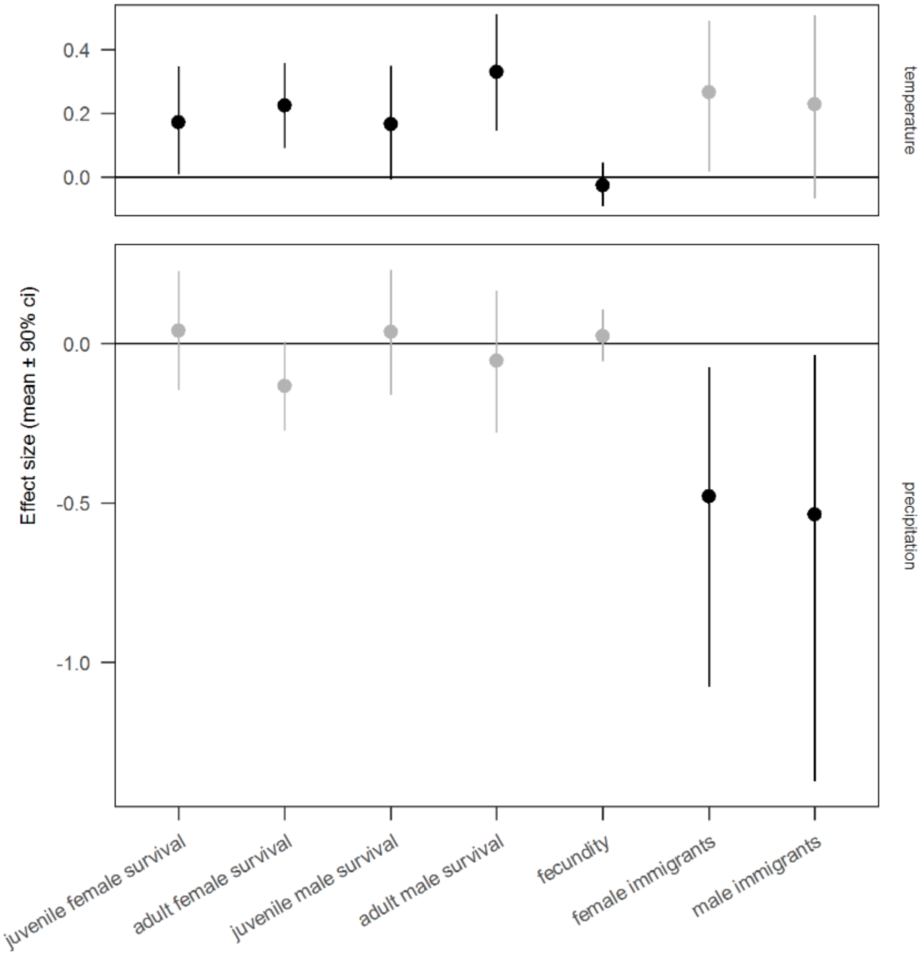

Supplementary Figure 6. Effects of weather at the wintering grounds on vital rates of Savannah sparrows from a breeding population on Kent Is., NB, Canada. Vital rates were modeled against standardized average daily mean temperatures and precipitation at the wintering grounds within an integrated population model. For a given vital rate, the strongest effect is shown in black and the weaker effect is shown in grey. Vital rates in year *t* were regressed against daily mean temperatures and precipitation averaged over the period of 01 Nov in year *t-1* to 31 Mar in year *t*. Temperature had strong positive effects on survival of adults and juveniles of both sexes (juvenile female = 0.17 [**95% CI =** 0.01, 0.35]; adult female = 0.22 [**95% CI =** 0.09, 0.36]; juvenile male = 0.17 [**95% CI =** -0.01, 0.35]; adult male = 0.33 [**95% CI =** 0.14, 0.51]) as well as on numbers of immigrants of both sexes (female = 0.27 [**95% CI =** 0.02, 0.49]; male = 0.23 [**95% CI =** -0.07, 0.50]). We also found evidence for immigration being negatively correlated with winter precipitation (female = -0.47 [**95% CI =** -1.08, -0.08]; male = -0.54 [**95% CI =** -1.37, -0.04]), but precision of the estimated slope coefficient was poor. Similar to the breeding grounds, fecundity was the most weakly correlated of the vital rates with weather at the wintering grounds (temperature effect = -0.03 [**95% CI =** -0.09, 0.04]; precipitation effect = 0.02 [**95% CI =** -0.06, 0.11]).

Supplementary Methods

R and BUGS code for IPM and path analysis

Integrated population model

IPM code was adapted from [1] and includes the following modifications:

1. Recapture probabilities were fixed to 0 for 2005-2007 (t = 18, 19, 20) when the population study was interrupted.
2. Goodness-of-fit tests were added for the Cormack-Jolly-Seber and Poisson regression models following [2].

```
# Specify working directory ------------------------------------------------

setwd('C:/...')

# Write model in JAGS language ---------------------------------------------

sink("ipm_null_gof.jags")
cat("
    model
    {

    ############################################################
    # Define the priors for parameters
    ############################################################

    # Initial population sizes
    rf ~ dnorm(10, 0.01)T(0,)
    Rf[1] <- round(rf)      # Local recruits (females)
    sf ~ dnorm(15, 0.01)T(0,)
    Sf[1] <- round(sf)      # Surviving adults (females)
    ifem ~ dnorm(25, 0.01)T(0,)
    If[1] <- round(ifem)    # Immigrants (females)

    rm ~ dnorm(10, 0.01)T(0,)
    Rm[1] <- round(rm)      # Local recruits (males)
    sm ~ dnorm(15, 0.01)T(0,)
    Sm[1] <- round(sm)      # Surviving adults (males)
    imale ~ dnorm(25, 0.01)T(0,)
    Im[1] <- round(imale)   # Immigrants (males)

    # Relationships for vital rates parameters
    for (t in 1:(ti-1)){
    logit(phijf[t]) <- logit.b0.phijf + epsilon[t,1] # Juvenile female survival
    logit(phiaf[t]) <- logit.b0.phiaf + epsilon[t,2] # Adult female survival
    logit(phijm[t]) <- logit.b0.phijm + epsilon[t,3] # Juvenile male survival
    logit(phiam[t]) <- logit.b0.phiam + epsilon[t,4] # Adult male survival
    log(fec[t]) <- log.b0.fec + epsilon[t,5] # Fecundity
    log(omegaf[t]) <- log.b0.omf + epsilon[t,6] # Female immigrants
```

```
    log(omegam[t]) <- log.b0.omm + epsilon[t,7] # Male immigrants
    }

    # Fix recapture probability to zero for lost years and
    # specify priors for recapture probability

    for (t in c(1:17, 21:(ti-1))){
    logit(pjf[t]) <- logit.b0.pjf + err.pjf[t]
    logit(pjm[t]) <- logit.b0.pjm + err.pjm[t]
    logit(pf[t]) <- logit.b0.pf + err.pf[t]
    logit(pm[t]) <- logit.b0.pm + err.pm[t]
    err.pjf[t] ~ dnorm(0, tau.pjf)
    err.pjm[t] ~ dnorm(0, tau.pjm)
    err.pf[t] ~ dnorm(0, tau.pf)
    err.pm[t] ~ dnorm(0, tau.pm)
    }

    for (t in 18:20){
    pjf[t] <- 0 ; err.pjf[t] <- 0
    pjm[t] <- 0 ; err.pjm[t] <- 0
    pf[t] <- 0 ; err.pf[t] <- 0
    pm[t] <- 0 ; err.pm[t] <- 0
    }

    # Priors for random effects of demographic rates
    for (i in 1:7) {zero[i] <- 0}
    for (t in 1:(ti-1)){
    epsilon[t,1:7]  ~ dmnorm(zero[], Omega[,])
    }

    # Prior for precision matrix
    Omega[1:7, 1:7]  ~ dwish(R[ , ], 8)
    Sigma[1:7, 1:7] <- inverse(Omega[ , ])

    # Priors for variance parameters (hyperparameters) of resighting rates and census
error
    tau.pjf <- 1 / pow(sigma.pjf, 2)
    sigma.pjf ~ dunif(0, 10)
    sigma2.pjf <- pow(sigma.pjf, 2)

    tau.pf <- 1 / pow(sigma.pf, 2)
    sigma.pf ~ dunif(0, 10)
    sigma2.pf <- pow(sigma.pf, 2)

    tau.pjm <- 1 / pow(sigma.pjm, 2)
    sigma.pjm ~ dunif(0, 10)
    sigma2.pjm <- pow(sigma.pjm, 2)

    tau.pm <- 1 / pow(sigma.pm, 2)
    sigma.pm ~ dunif(0, 10)
    sigma2.pm <- pow(sigma.pm, 2)

    tau.c ~ dgamma(0.001, 0.001)
    sigma2.c <- 1 / tau.c
```

```
    # Priors for the mean of demographic and resighting rates
    b0.phijf ~ dunif(0, 1)
    b0.phiaf ~ dunif(0, 1)
    b0.phijm ~ dunif(0, 1)
    b0.phiam ~ dunif(0, 1)
    b0.fec ~ dunif(0, 10)
    b0.omf ~ dunif(0, 75)
    b0.omm ~ dunif(0, 75)
    b0.pjf ~ dunif(0, 1)
    b0.pf ~ dunif(0, 1)
    b0.pjm ~ dunif(0, 1)
    b0.pm ~ dunif(0, 1)

    # Back-transformations
    logit.b0.phijf <- log(b0.phijf / (1 - b0.phijf))
    logit.b0.phiaf <- log(b0.phiaf / (1 - b0.phiaf))
    logit.b0.phijm <- log(b0.phijm / (1 - b0.phijm))
    logit.b0.phiam <- log(b0.phiam / (1 - b0.phiam))
    log.b0.fec <- log(b0.fec)
    log.b0.omf <- log(b0.omf)
    log.b0.omm <- log(b0.omm)
    logit.b0.pjf <- log(b0.pjf / (1 - b0.pjf))
    logit.b0.pf <- log(b0.pf / (1 - b0.pf))
    logit.b0.pjm <- log(b0.pjm / (1 - b0.pjm))
    logit.b0.pm <- log(b0.pm / (1 - b0.pm))

    # Prior for productivity in the last year
    fec[ti] ~ dunif(0, 10)   # treated as fixed effect (not possible to be included i
n random effects)

    ######################################################################
    # Likelihoods of the integrated population model
    ######################################################################

    ####################################################
    # - Likelihood for reproductive data
    ####################################################

    for (t in 1:ti){
    J[t] ~ dpois(M[t] * fec[t])
    J.rep[t] ~ dpois(M[t] * fec[t]) # replicate data for GOF

    # Compute statistics for posterior predictive checks
    J.exp[t] <- M[t] * fec[t]
    J.chi[t] <- (J[t] - J.exp[t])^2 / (J.exp[t] + 0.5)
    J.chi.new[t] <- (J.rep[t] - J.exp[t])^2 / (J.exp[t] + 0.5)
    } #i

    fit.J <- sum(J.chi[1:ti])
    fit.J.new <- sum(J.chi.new[1:ti])

    ####################################################
    # - Likelihood for population survey data
    ####################################################
```

```
    ##############################
    # -- System process
    ##############################

    for (t in 2:ti){
    meanfl[t-1] <- fec[t-1] * Bf[t-1]
    F[t-1] ~ dpois(meanfl[t-1])          # Total number of fledglings
    Ff[t-1] ~ dbin(0.5, F[t-1])          # Number of female fledglings
    Rf[t] ~ dbin(phijf[t-1], Ff[t-1])    # Number of female local recruits
    Sf[t] ~ dbin(phiaf[t-1], Bf[t-1])    # Number of surviving adult females
    If[t] ~ dpois(omegaf[t-1])           # Number of immigrated females

    Fm[t-1] <- F[t-1] - Ff[t-1]          # Number of male fledglings
    Rm[t] ~ dbin(phijm[t-1], Fm[t-1])    # Number of male local recruits
    Sm[t] ~ dbin(phiam[t-1], Bm[t-1])    # Number of surviving adult males
    Im[t] ~ dpois(omegam[t-1])           # Number of immigrated males
    } # t

    # Number of fledglings in the last study year
    meanfl[ti] <- fec[ti] * Bf[ti]
    F[ti] ~ dpois(meanfl[ti])
    Ff[ti] ~ dbin(0.5, F[ti])
    Fm[ti] <- F[ti] - Ff[ti]


    ################################
    # -- Observation process
    ################################

    for(t in 1:ti){
    Bf[t] <- Sf[t] + Rf[t] + If[t]       # Total number of breeding females
    logBf[t] <- log(Bf[t])
    Cf[t] ~ dlnorm(logBf[t], tau.c)

    Bm[t] <- Sm[t] + Rm[t] + Im[t]       # Total number of breeding males
    logBm[t] <- log(Bm[t])
    Cm[t] ~ dlnorm(logBm[t], tau.c)
    } # t

    ################################################
    # - Likelihood for capture-recapture data (Cormack-Jolly-Seber model with 2 age c
lasses)
    ################################################

    # Likelihood
    for (t in 1:(2*(ti-1))) {
    MF[t,1:ti] ~ dmulti(prf[t,], r.f[t])
    MM[t,1:ti] ~ dmulti(prm[t,], r.m[t])
    } # t

    # m-array cell probabilities for juveniles
    for (t in 1:(ti-1)){
    qf[t] <- 1-pf[t]        # probability of non-capture (females)
    qm[t] <- 1-pm[t]        # probability of non-capture (males)
    # main diagonal
```

```
    prf[t,t] <- phijf[t] * pjf[t]
    prm[t,t] <- phijm[t] * pjm[t]

    # above main diagonal
    for (j in (t+1):(ti-1)){
    prf[t,j] <- phijf[t] * prod(phiaf[(t+1):j]) * (1-pjf[t]) * prod(qf[t:(j-1)]) * pf
[j] / qf[t]
    prm[t,j] <- phijm[t] * prod(phiam[(t+1):j]) * (1-pjm[t]) * prod(qm[t:(j-1)]) * pm
[j] / qm[t]
    } # j

    # below main diagonal
    for (j in 1:(t-1)){
    prf[t,j] <- 0
    prm[t,j] <- 0
    } # j

    # last column
    prf[t,ti] <- 1 - sum(prf[t,1:(ti-1)])
    prm[t,ti] <- 1 - sum(prm[t,1:(ti-1)])
    } # t

    # m-array cell probabilities for adults
    for (t in 1:(ti-1)){
    # main diagonal
    prf[t+ti-1,t] <- phiaf[t] * pf[t]
    prm[t+ti-1,t] <- phiam[t] * pm[t]

    # above main diagonal
    for (j in (t+1):(ti-1)){
    prf[t+ti-1,j] <- prod(phiaf[t:j]) * prod(qf[t:(j-1)]) * pf[j]
    prm[t+ti-1,j] <- prod(phiam[t:j]) * prod(qm[t:(j-1)]) * pm[j]
    } # j

    # below main diagonal
    for (j in 1:(t-1)){
    prf[t+ti-1,j] <- 0
    prm[t+ti-1,j] <- 0
    } # j

    # last column
    prf[t+ti-1,ti] <- 1 - sum(prf[t+ti-1,1:(ti-1)])
    prm[t+ti-1,ti] <- 1 - sum(prm[t+ti-1,1:(ti-1)])
    } # t

    # Compute Freeman-Tukey statistics for posterior predictive checks of the CR data
    for (t1 in 1:(ti-1)) {
    for (t2 in 1:ti) {
    # Juveniles
    exp.Mjf[t1,t2] <- prf[t1,t2] * r.f[t1]
    exp.Mjm[t1,t2] <- prm[t1,t2] * r.m[t1]
    E.org.jf[t1,t2] <- pow((pow(MF[t1,t2],0.5)-pow(exp.Mjf[t1,t2],0.5)),2)
    E.org.jm[t1,t2] <- pow((pow(MM[t1,t2],0.5)-pow(exp.Mjm[t1,t2],0.5)),2)
    # Adults
    exp.Maf[t1,t2] <- prf[t1+ti-1,t2] * r.f[t1+ti-1]
```

```
    exp.Mam[t1,t2] <- prm[t1+ti-1,t2] * r.m[t1+ti-1]
    E.org.af[t1,t2] <- pow((pow(MF[t1+ti-1,t2],0.5)-pow(exp.Maf[t1,t2],0.5)),2)
    E.org.am[t1,t2] <- pow((pow(MM[t1+ti-1,t2],0.5)-pow(exp.Mam[t1,t2],0.5)),2)
    } # t2
    } # t1

    # Generate replicate data and compute fit statistics
    for (t1 in 1:(ti-1)){
    # Juveniles
    new.Mjf[t1,1:ti] ~ dmulti(prf[t1,], r.f[t1])
    new.Mjm[t1,1:ti] ~ dmulti(prm[t1,], r.m[t1])
    # Adults
    new.Maf[t1,1:ti] ~ dmulti(prf[t1+ti-1,], r.f[t1+ti-1])
    new.Mam[t1,1:ti] ~ dmulti(prm[t1+ti-1,], r.m[t1+ti-1])
    for (t2 in 1:ti){
    # Juveniles
    E.new.jf[t1,t2] <- pow((pow(new.Mjf[t1,t2],0.5)-pow(exp.Mjf[t1,t2],0.5)),2)
    E.new.jm[t1,t2] <- pow((pow(new.Mjm[t1,t2],0.5)-pow(exp.Mjm[t1,t2],0.5)),2)
    # Adults
    E.new.af[t1,t2] <- pow((pow(new.Maf[t1,t2],0.5)-pow(exp.Maf[t1,t2],0.5)),2)
    E.new.am[t1,t2] <- pow((pow(new.Mam[t1,t2],0.5)-pow(exp.Mam[t1,t2],0.5)),2)
    } # t2
    } # t1

    # Summarize test statistics for each age-sex group
    fit.jf <- sum(E.org.jf[,])
    fit.jm <- sum(E.org.jm[,])
    fit.new.jf <- sum(E.new.jf[,])
    fit.new.jm <- sum(E.new.jm[,])
    fit.af <- sum(E.org.af[,])
    fit.am <- sum(E.org.am[,])
    fit.new.af <- sum(E.new.af[,])
    fit.new.am <- sum(E.new.am[,])

    }  # End Model
    ",fill = TRUE)
sink()

# Specify required data for the model ----------------------------------------

load('ipm_data.RData')

# M, BF = vectors of annual counts of breeding females
# BM = vector of annual counts of breeding males
# J = vector of annual numbers of fledged young
# MF = matrix of female capture-recapture/resighting data in multinomial array format
# MM = matrix of male capture-recapture/resighting data in multinomial array format

data =
  list(ti = ncol(MF),
       MF = MF, MM = MM,
       r.f = rowSums(MF), r.m = rowSums(MM),
       Cf = BF[1:ncol(MF)], Cm = BM[1:ncol(MM)],
       J = J[1:ncol(MF)], M = M[1:ncol(MF)],
       R = diag(c(1,1,1,1,1,1,1), ncol = 7))
```

```r
# Run the model in JAGS from R using jagsUI package --------------------------

library(jagsUI)

# MCMC settings
chain = 3
burn = 500000
iter = 1000000
thin = 100

# Specify initial values
ti = ncol(MF)

inits =
  function() {
    list(b0.phijf = runif(1,0,0.5), b0.phiaf = runif(1,0.1,0.6),
         b0.phijm = runif(1,0,0.5), b0.phiam = runif(1,0.1,0.6),
         b0.fec = runif(1,2,3), b0.omf = runif(1,0,20), b0.omm = runif(1,0,20),
         b0.pjf = runif(1,0.2,0.7), b0.pf = runif(1,0.1,0.6), b0.pjm = runif(1,0.2,0.
7), b0.pm = runif(1,0.5,1),
         sigma.pjf = runif(1,0,1), sigma.pf = runif(1,0,1), sigma.pjm = runif(1,0,1),
sigma.pm = runif(1,0,1),
         If = c(NA, round(runif(ti-1,0,20),0)),
         Im = c(NA, round(runif(ti-1,0,20),0)),
         Omega = diag(7))}

# Define parameters to be monitored
parameters =
  c("phijf", "phiaf", "phijm", "phiam",
    "b0.phijf", "b0.phiaf", "b0.phijm", "b0.phiam",
    "pf", "pjf", "pm", "pjm",
    "b0.pjf", "b0.pf", "b0.pjm", "b0.pm",
    "fec", "b0.fec", "F",
    'omegam','omegaf',"b0.omf", "b0.omm",
    "Sigma", "sigma2.pjf", "sigma2.pf",
    "iratef","iratem","irate",
    "sigma2.pjm", "sigma2.pm", "sigma2.c",
    "Rf", "Sf", "Bf", "If", "Ff",
    "Rm", "Sm", "Bm", "Im", "Fm",
    "fit.jf","fit.new.jf","fit.jm","fit.new.jm",
    "fit.af","fit.new.af","fit.am","fit.new.am",
    "fit.J", "fit.J.new")

# Run the model
ipm_gof =
  jags(data, inits = inits, model.file = "ipm_null_gof.jags", parameters = parameters
,
       n.iter = iter, n.chains = chain, n.burnin = burn, n.thin = thin,
       parallel = TRUE)

# View and save model results
print(ipm_gof, digits = 3)

save(ipm_gof, file = 'ipm_null_gof.RData')
```

Variable selection

To reduce the set of variables considered in the final path model of factors limiting and regulating population growth rate, we conducted a variable selection procedure that involved fitting each vital rate as a linear function of a single weather or density variable from a given period of the annual cycle. Code for fitting univariable models is given below.

```
# Write models in JAGS language ---------------------------------------------

### Univariable models for survival

sink("ipm_univ_phi.jags")
cat("
    model
    {

    ### ... (model code up to here is the same as ipm_null_gof.jags) ...

    # Relationships for vital rates parameters
    for (t in 1:(ti-1)){
    logit(phijf[t]) <- logit.b0.phijf + b[1]*cov[t] + epsilon[t,1]
    logit(phiaf[t]) <- logit.b0.phiaf + b[2]*cov[t] + epsilon[t,2]
    logit(phijm[t]) <- logit.b0.phijm + b[3]*cov[t] + epsilon[t,3]
    logit(phiam[t]) <- logit.b0.phiam + b[4]*cov[t] + epsilon[t,4]
    log(fec[t]) <- log.b0.fec + epsilon[t,5]
    log(omegaf[t]) <- log.b0.omf + epsilon[t,6]
    log(omegam[t]) <- log.b0.omm + epsilon[t,7]
    }

    # Priors for regression coefficients
    for (i in 1:4) { b[i] ~ dnorm(0, 0.0001)T(-5,5) }

    # For models of breeding density-dependence, we specified priors for breeding den
sities
    # from 2005 (t=19) to 2007 (t=21) when the population study was interrupted,
    # where dd.prior equals the mean of breeding densities at t=18 and t=22

    # for (t in 19:21) { cov[t] ~ dnorm(dd.prior, 1) }

    ### ... (model code hereafter is the same as ipm_null_gof.jags) ...

    }  # End Model
    ",fill = TRUE)
sink()

### Univariable models for fecundity

sink("ipm_univ_fec.jags")
cat("
    model
```

```
    {

    ### ... (model code up to here is the same as ipm_null_gof.jags) ...

    # Relationships for vital rates parameters
    for (t in 1:(ti-1)){
    logit(phijf[t]) <- logit.b0.phijf + epsilon[t,1]
    logit(phiaf[t]) <- logit.b0.phiaf + epsilon[t,2]
    logit(phijm[t]) <- logit.b0.phijm + epsilon[t,3]
    logit(phiam[t]) <- logit.b0.phiam + epsilon[t,4]
    log(fec[t]) <- log.b0.fec + b[1]*cov[t] + epsilon[t,5]
    log(omegaf[t]) <- log.b0.omf + epsilon[t,6]
    log(omegam[t]) <- log.b0.omm + epsilon[t,7]
    }

    # Priors for regression coefficients
    for (i in 1) { b[i] ~ dnorm(0, 0.0001)T(-5,5) }

    # For models of breeding density-dependence, we specified priors for breeding den
sities
    # from 2005 (t=19) to 2007 (t=21) when the population study was interrupted,
    # where dd.prior equals the mean of breeding densities at t=18 and t=22

    # for (t in 19:21) { cov[t] ~ dnorm(dd.prior, 1) }

    ### ... (model code hereafter is the same as ipm_null_gof.jags) ...

    }  # End Model
    ",fill = TRUE)
sink()

### Univariable models for immigration

sink("ipm_univ_omega.jags")
cat("
    model
    {

    ### ... (model code up to here is the same as ipm_null_gof.jags) ...

    # Relationship for vital rates parameters
    for (t in 1:(ti-1)){
    logit(phijf[t]) <- logit.b0.phijf + epsilon[t,1]
    logit(phiaf[t]) <- logit.b0.phiaf + epsilon[t,2]
    logit(phijm[t]) <- logit.b0.phijm + epsilon[t,3]
    logit(phiam[t]) <- logit.b0.phiam + epsilon[t,4]
    log(fec[t]) <- log.b0.fec + epsilon[t,5]
    log(omegaf[t]) <- log.b0.omf + b[1]*cov[t] + epsilon[t,6]
    log(omegam[t]) <- log.b0.omm + b[2]*cov[t] + epsilon[t,7]
    }

    # Priors for regression coefficients
    for (i in 1:2) { b[i] ~ dnorm(0, 0.0001)T(-5,5) }

    ### ... (model code hereafter is the same as ipm_null_gof.jags) ...
```

```
    }  # End Model
    ",fill = TRUE)
sink()

# Define parameters for running models in loop (see below) -----------------

model = one of 'ipm_univ_phi.jags' or 'ipm_univ_fec.jags' or 'ipm_univ_omega.jags'
nbeta = number of regression coefficients estimated in model

# Load covariate data ---------------------------------------------------

load('ipm_covariate_data.RData')

### stdBa = vector of standardized breeding densities for survival models
### stdBa.dt = vector of standardized, de-trended breeding densities for fecundity mo
dels
### stdNBa = vector of standardized winter densities
### mean_ssn_temp_phi, mean_ssn_precip_phi =
###        matrices of pre-breeding, breeding, and post-breeding average daily mean
###        temperatures and precipitation at the breeding grounds for survival models
### mean_ssn_temp_fec, mean_ssn_precip_fec =
###        matrices of pre-breeding, breeding, and post-breeding average daily mean
###        temperatures and precipitation at the breeding grounds for fecundity model
s
### mean_nb_temp = matrix of average daily mean temperatures at the wintering grounds
### mean_nb_precip = matrix of average daily precipitation at the wintering grounds

cov.mat = one of above vectors or matrices of annual density or weather values

### For estimating breeding density-dependence:
dd.prior = mean(c(stdBa[18], stdBa[22])) # for survival models
### OR
dd.prior = mean(c(stdBa.dt[18], stdBa.dt[22])) # for fecundity models

# Specify MCMC settings and generate initial values ------------------------

### MCMC settings
chain = 3
burn = 50000
iter = 100000
thin = 10

### Initial values
ti = ncol(MF)

inits <-
  function() {
    list(b0.phijf = runif(1,0,0.5), b0.phiaf = runif(1,0.1,0.6),
         b0.phijm = runif(1,0,0.5), b0.phiam = runif(1,0.1,0.6),
         b0.pjf = runif(1,0.2,0.7), b0.pf = runif(1,0.1,0.6),
         b0.pjm = runif(1,0.2,0.7), b0.pm = runif(1,0.5,1),
         sigma.pjf = runif(1,0,1), sigma.pf = runif(1,0,1),
         sigma.pjm = runif(1,0,1), sigma.pm = runif(1,0,1),
         b0.omf = runif(1,0,20), b0.omm = runif(1,0,20),
```

```
        If = c(NA, round(runif(ti-1,0,20),0)),
        Im = c(NA, round(runif(ti-1,0,20),0)),
        b0.fec = runif(1,2,3),
        Omega = diag(7),
        b = rnorm(nbeta, 0.2,0.5))} # initial values for regression coefficients

### Define parameters to be monitored
parameters <-
  c("phijf", "phiaf", "phijm", "phiam",
    "b0.phijf", "b0.phiaf", "b0.phijm", "b0.phiam",
    "pf", "pjf", "pm", "pjm",
    "b0.pjf", "b0.pf", "b0.pjm", "b0.pm",
    "fec", "b0.fec", "F",
    'omegam','omegaf',"b0.omm", "b0.omf",
    "Sigma", "sigma2.pjf", "sigma2.pf",
    "sigma2.pjm", "sigma2.pm", "sigma2.c",
    "Rf", "Sf", "Bf", "If", "Ff",
    "Rm", "Sm", "Bm", "Im", "Fm",
    "fit.jf","fit.new.jf","fit.jm","fit.new.jm",
    "fit.af","fit.new.af","fit.am","fit.new.am",
    "fit.J", "fit.J.new",
    "b") # monitor regression coefficients

# Run models in loop and save output---------------------------------------

ipm = NULL
start = Sys.time()
for (z in 1:ncol(cov.mat)) {

  data =
    list(ti = ncol(MF),
         MF = MF, MM = MM,
         r.f = rowSums(MF), r.m = rowSums(MM),
         Cf = BF[1:ncol(MF)], Cm = BM[1:ncol(MF)],
         J = J[1:ncol(MF)], M = M[1:ncol(MF)],
         R = diag(c(1,1,1,1,1,1,1), ncol = 7),
         dd.prior = dd.prior, # only used for breeding density-dependence models
         cov = cov.mat[,z])

  ipm =
    jags(data, inits = inits, model.file = model, parameters = parameters,
         n.iter = iter, n.chains = chain, n.burnin = burn, n.thin = thin,
         parallel = TRUE)

  save(ipm, file = paste0(paste(substr(model, 1, 12), z, sep="_"), ".RData"))

  print(z)

}
end = Sys.time()
end - start
```

Path analysis

Below we provide code for the path analysis to quantify the relative effects of weather and density at the breeding and wintering grounds on population growth rate via the vital rates. The path model was fitted to annual estimates of vital rates and population growth rate from the null IPM and key weather and density variables identified from the univariable models described above.

```
load('ipm_null_gof.RData')

# Create vectors and matrices for storing direct and indirect effects ------

### *.mat and *.r2 objects contain standardized regression coefficients and
### r-squared values, respectively, from models of population growth rate vs.
### vital rates OR vital rates vs. weather and density

### *.ie.* objects denote vectors of indirect effects of a given weather or
### density covariate on population growth rate (lambda). As an example,
### phijf.ie.X is a vector of indirect effects of variable X on lambda via
### phijf, whereas ie.X is a vector of the cumulative indirect effects of
### variable X on lambda across all vital rates. Weather and density variables
### are denoted as follows:
###   NBt = winter temperature
###   NBp = winter precipitation
###   Bt = breeding temperature
###   Bp = breeding precipitation
###   preBt = pre-breeding temperature
###   preBp = pre-breeding precipitation
###   postBt = post-breeding temperature
###   postBp = post-breeding precipitation
###   Bdd = breeding density-dependence
###   NBdd = winter density-dependence

n.samples = ipm_gof$mcmc.info$n.samples

### Population growth rate

##### Total immigration rate
lambda.mat = matrix(nrow = n.samples, ncol = 6)
lambda.r2 = vector(length = n.samples)

##### Sex-specific immigration rate
lambda.ssim.mat = matrix(nrow = n.samples, ncol = 7)
lambda.ssim.r2 = vector(length = n.samples)

### Juvenile female survival
phijf.mat = matrix(nrow = n.samples, ncol = 3)
phijf.r2 = vector(length = n.samples)
phijf.ie.NBt = matrix(nrow = n.samples, ncol = 1)
phijf.ie.Bp = matrix(nrow = n.samples, ncol = 1)
phijf.ie.Bdd = matrix(nrow = n.samples, ncol = 1)
```

```r
### Adult female survival
phiaf.mat = matrix(nrow = n.samples, ncol = 3)
phiaf.r2 = vector(length = n.samples)
phiaf.ie.NBt = matrix(nrow = n.samples, ncol = 1)
phiaf.ie.postBt = matrix(nrow = n.samples, ncol = 1)
phiaf.ie.NBdd = matrix(nrow = n.samples, ncol = 1)

### Juvenile male survival
phijm.mat = matrix(nrow = n.samples, ncol = 3)
phijm.r2 = vector(length = n.samples)
phijm.ie.NBt = matrix(nrow = n.samples, ncol = 1)
phijm.ie.Bp = matrix(nrow = n.samples, ncol = 1)
phijm.ie.Bdd = matrix(nrow = n.samples, ncol = 1)

### Adult male survival
phiam.mat = matrix(nrow = n.samples, ncol = 3)
phiam.r2 = vector(length = n.samples)
phiam.ie.NBt = matrix(nrow = n.samples, ncol = 1)
phiam.ie.preBt = matrix(nrow = n.samples, ncol = 1)
phiam.ie.Bdd = matrix(nrow = n.samples, ncol = 1)

### Fecundity
fec.mat = matrix(nrow = n.samples, ncol = 3)
fec.r2 = vector(length = n.samples)
fec.ie.Bt = matrix(nrow = n.samples, ncol = 1)
fec.ie.NBt = matrix(nrow = n.samples, ncol = 1)
fec.ie.Bdd = matrix(nrow = n.samples, ncol = 1)

### Immigration
im.mat = matrix(nrow = n.samples, ncol = 2)
im.r2 = vector(length = n.samples)
im.ie.NBp = matrix(nrow = n.samples, ncol = 1)
im.ie.NBt = matrix(nrow = n.samples, ncol = 1)

### Indirect effects
ie.Bdd = matrix(nrow = n.samples, ncol = 1)
ie.NBdd = matrix(nrow = n.samples, ncol = 1)
ie.NBt = matrix(nrow = n.samples, ncol = 1)
ie.NBp = matrix(nrow = n.samples, ncol = 1)
ie.Bp = matrix(nrow = n.samples, ncol = 1)
ie.Bt = matrix(nrow = n.samples, ncol = 1)
ie.postBt = matrix(nrow = n.samples, ncol = 1)
ie.preBt = matrix(nrow = n.samples, ncol = 1)

# Run path model ---------------------------------------------------------

### We excluded population growth rate and vital rate estimates from the years the
### population study was interrupted (2005-2007).

for (i in 1:n.samples) {

  # Calculate population growth rate and immigration rates from IPM estimates
  # of sex- and stage-specific abundances

  lambda = vector(length = 28) # Population growth rate
```

```
imf = vector(length = 28) # Female immigration rate
imm = vector(length = 28) # Male immigration rate
im = vector(length = 28) # Total immigration rate

for (t in 1:28){
  lambda[t] <-
    (ipm_gof$sims.list$Bf[i,t+1] + ipm_gof$sims.list$Bm[i,t+1]) /
    (ipm_gof$sims.list$Bf[i,t] + ipm_gof$sims.list$Bm[i,t])

  imf[t] =
    (ipm_gof$sims.list$If[i,t+1])/ (ipm_gof$sims.list$Bf[i,t])

  imm[t] =
    ipm_gof$sims.list$Im[i,t+1] / (ipm_gof$sims.list$Bm[i,t])

  im[t] =
    (ipm_gof$sims.list$Im[i,t+1] + ipm_gof$sims.list$If[i,t+1]) /
    (ipm_gof$sims.list$Bm[i,t] + ipm_gof$sims.list$Bf[i,t])
  }

# Direct effects of vital rates on lambda

### Total immigration rate
lambda.lm =
  lm(scale(lambda[-c(18:21)]) ~
       scale(ipm_gof$sims.list$phijf[i,-c(18:21)]) +
       scale(ipm_gof$sims.list$phiaf[i,-c(18:21)]) +
       scale(ipm_gof$sims.list$phijm[i,-c(18:21)]) +
       scale(ipm_gof$sims.list$phiam[i,-c(18:21)]) +
       scale(ipm_gof$sims.list$fec[i,-c(18:21,29)]) +
       scale(im[-c(18:21)]))

lambda.mat[i,] = lambda.lm$coefficients[-1]
lambda.r2[i] = summary(lambda.lm)$r.squared

### Sex-specific immigration rates
lambda.ssim.lm =
  lm(scale(lambda[-c(18:21)]) ~
       scale(ipm_gof$sims.list$phijf[i,-c(18:21)]) +
       scale(ipm_gof$sims.list$phiaf[i,-c(18:21)]) +
       scale(ipm_gof$sims.list$phijm[i,-c(18:21)]) +
       scale(ipm_gof$sims.list$phiam[i,-c(18:21)]) +
       scale(ipm_gof$sims.list$fec[i,-c(18:21,29)]) +
       scale(imf[-c(18:21)]) +
       scale(imm[-c(18:21)]))

lambda.ssim.mat[i,] = lambda.ssim.lm$coefficients[-1]
lambda.ssim.mat[i] = summary(lambda.ssim.lm)$r.squared

# Direct effects of weather and density on vital rates

### Juvenile female survival
phijf.lm =
  lm(scale(ipm_gof$sims.list$phijf[i,-c(18:21)]) ~
       mean_ssn_precip_phi[-c(18:21),'breeding'] +
```

```
        mean_nb_temp[-c(1,18:21),'Nov-Mar'] +
        stdBa[-c(18:21,29)])

phijf.mat[i,] = phijf.lm$coefficients[-1]
phijf.r2[i] = summary(phijf.lm)$r.squared

##### Indirect effects on lambda via phijf
phijf.ie.Bp[i] = phijf.mat[i,1] * lambda.mat[i,1]
phijf.ie.NBt[i] = phijf.mat[i,2] * lambda.mat[i,1]
phijf.ie.Bdd[i] = phijf.mat[i,3] * lambda.mat[i,1]

### Adult female survival
phiaf.lm =
  lm(scale(ipm_gof$sims.list$phiaf[i,-c(18:21)]) ~
        mean_ssn_temp_phi[-c(18:21),'post-breeding'] +
        mean_nb_temp[-c(1,18:21),'Nov-Mar'] +
        stdNBa$std_count[-c(-1,18:21)])

phiaf.mat[i,] = phiaf.lm$coefficients[-1]
phiaf.r2[i] = summary(phiaf.lm)$r.squared

##### Indirect effects on lambda via phiaf
phiaf.ie.postBt[i] = phiaf.mat[i,1] * lambda.mat[i,2]
phiaf.ie.NBt[i] = phiaf.mat[i,2] * lambda.mat[i,2]
phiaf.ie.NBdd[i] = phiaf.mat[i,3] * lambda.mat[i,2]

### Juvenile male survival
phijm.lm =
  lm(scale(ipm_gof$sims.list$phijm[i,-c(18:21)]) ~
        mean_ssn_precip_phi[-c(18:21),'breeding'] +
        mean_nb_temp[-c(1,18:21),'Nov-Mar']+
        stdBa[-c(18:21,29)])

phijm.mat[i,] = phijm.lm$coefficients[-1]
phijm.r2[i] = summary(phijm.lm)$r.squared

##### Indirect effects on lambda via phijm
phijm.ie.Bp[i] = phijm.mat[i,1] * lambda.mat[i,3]
phijm.ie.NBt[i] = phijm.mat[i,2] * lambda.mat[i,3]
phijm.ie.Bdd[i] = phijm.mat[i,3] * lambda.mat[i,3]

### Adult male survival
phiam.lm =
  lm(scale(ipm_gof$sims.list$phiam[i,-c(18:21)]) ~
        mean_ssn_temp_phi[-c(18:21),'pre-breeding'] +
        mean_nb_temp[-c(1,18:21),'Nov-Mar'] +
        stdBa[-c(18:21,29)])

phiam.mat[i,] = phiam.lm$coefficients[-1]
phiam.r2[i] = summary(phiam.lm)$r.squared

##### Indirect effects on lambda via phiam
phiam.ie.preBt[i] = phiam.mat[i,1] * lambda.mat[i,4]
phiam.ie.NBt[i] = phiam.mat[i,2] * lambda.mat[i,4]
phiam.ie.Bdd[i] = phiam.mat[i,3] * lambda.mat[i,4]
```

```
  ### Fecundity
  fec.lm =
    lm(scale(ipm_gof$sims.list$fec[i,-c(19:21,29)]) ~
         mean_ssn_temp_fec[-c(19:21, 29),'breeding'] +
         mean_nb_temp[-c(19:21,29),'Nov-Mar'] +
         stdBa.dt[-c(19:21,29)])

  fec.mat[i,] = fec.lm$coefficients[-1]
  fec.r2[i] = summary(fec.lm)$r.squared

  ##### Indirect effects on lambda via fecundity
  fec.ie.Bt[i] = fec.mat[i,1] * lambda.mat[i,5]
  fec.ie.NBt[i] = fec.mat[i,2] * lambda.mat[i,5]
  fec.ie.Bdd[i] = fec.mat[i,3] * lambda.mat[i,5]

  ### Immigration rate
  im.lm =
    lm(scale(im[-c(18:21)]) ~
         mean_nb_temp[-c(1,18:21),'Nov-Mar'] +
         mean_nb_precip[-c(1,18:21),'Nov-Mar']
       )

  im.mat[i,] = im.lm$coefficients[-1]
  im.r2[i] = summary(im.lm)$r.squared

  ##### Indirect effects on lambda via immigration rate
  im.ie.NBt[i] = im.mat[i,1] * lambda.mat[i,6]
  im.ie.NBp[i] = im.mat[i,2] * lambda.mat[i,6]

  # Cumulative indirect effects of covariates on lambda via survival and fecundity

  ### Breeding density-dependence
  ie.Bdd[i] = phijf.ie.Bdd[i] + phijm.ie.Bdd[i] + phiam.ie.Bdd[i] + fec.ie.Bdd[i]
  ### Winter density-dependence
  ie.NBdd[i] = phiaf.ie.NBdd[i]
  ### Winter temperature
  ie.NBt[i] =
    phijf.ie.NBt[i] + phiaf.ie.NBt[i] + phijm.ie.NBt[i] + phiam.ie.NBt[i] + fec.ie.NB
t[i] +
    im.ie.NBt[i]
  ### Winter precipitation
  ie.NBp[i] = im.ie.NBp[i]
  ### Breeding temperature
  ie.Bt[i] = fec.ie.Bt[i]
  ### Breeding precipitation
  ie.Bp[i] =  phijf.ie.Bp[i] + phijm.ie.Bp[i]
  ### Pre-breeding temperature
  ie.preBt[i] = phiam.ie.preBt[i]
  ### Post-breeding temperature
  ie.postBt[i] = phiaf.ie.postBt[i]

  }

# Save model output ------------------------------------------------------------
```

```r
save(list = c(ls()[grep(ls(), pattern = 'ie.')],
              ls()[grep(ls(), pattern = 'mat')],
              ls()[grep(ls(), pattern = 'r2')],
              'lambda.mat'),
     file = 'path-results.RData')
```

Supplementary References

1. Schaub, M., Jakober, H. & Stauber, W. Strong contribution of immigration to local population regulation: evidence from a migratory passerine. *Ecology*. **94**, 1828–1838 (2013).

2. Schaub, M, von Hirschheydt, J. & Grüebler, M. U. Differential contribution of demographic rate synchrony to population synchrony in barn swallows. *J. Anim. Ecol.* **84**, 1530–1541 (2015).
